# Bioremediation of oil contaminated soil using agricultural wastes via microbial consortium

**DOI:** 10.1038/s41598-020-66169-5

**Published:** 2020-06-08

**Authors:** Chao Zhang, Daoji Wu, Huixue Ren

**Affiliations:** 1 School of Municipal and Environmental Engineering, Shandong Jianzhu University, JiNan, 250101 China; 2 Co-Innovation Center of Green Building, JiNan, 250101 China

**Keywords:** Environmental biotechnology, Soil microbiology

## Abstract

Agricultural wastes, such as wheat bran and swine wastewater, were used for bioremediation of oil-contaminated soil. Two optimised strains that could degrade oil efficiently were selected. The result showed that the best ratio of strain A to strain B was 7:3. Swine wastewater could be a replacement for nitrogen source and process water for bioremediation. Next, the Box-Behnken design was used to optimise the culture medium, and the optimal medium was as follows: microbial dosage of 97 mL/kg, wheat bran of 158 g/kg and swine wastewater of 232 mL/kg. Under the optimal medium, the oil degradation rate reached 68.27 ± 0.71% after 40 d. The urease, catalase, and dehydrogenase activities in oil-contaminated soil all increased, and the microbe quantity increased significantly with manual composting. These investigations might lay a foundation for reducing the pollution of agricultural wastes, exploring a late model for bioremediation of oil-contaminated soil.

## Introduction

A large number of oil pollutants are produced during the production, processing, transportation, and utilisation of oil^1,2^. This has caused serious soil pollution. Therefore, it is urgent to repair the oil-contaminated soil. Compared with physical repair and chemical repair, bioremediation has been widely used because of its advantages of good effect, easy operation, low cost, rapid degradation rate, and lack of secondary pollution^3,4^.

Bioremediation is divided into two types of remediation: *in-situ* and *ex-situ*. *In-situ* remediation techniques include land tillage, microorganism addition, bio-culture, and bio-ventilation. *Ex-situ* remediation technologies include prefabricated bed and bioreactor^5,6^. For example, Besalatpour et al^7^. used land tillage to remediate 500 kg of oil-contaminated soil in farmland. After four months, total petroleum hydrocarbons decreased by 50%. The essence of bioremediation technology is the degradation of pollutants through microbial metabolic activities. A majority of the microorganisms used in the process of remediation are indigenous microorganisms^8^. In order to improve the repair effect, domesticated high-efficiency oil-degrading bacteria were introduced. Wu et al^9^. repaired the contaminated soil with 82,533 mg/kg petroleum hydrocarbon content by adding mixed degrading bacteria. After 13 weeks, the content of petroleum hydrocarbon decreased to 47,600 mg/kg, and the removal rate of petroleum hydrocarbon reached 42.3%. Although there are many scholars engaged in this field, there remains a lack of low-cost and positive-effect technology. Existing investigations reported that the expensive refined supplements were still necessary for bioremediation, such as refined carbon source, refined nitrogen source, process water, or surface active agent. Therefore, the high cost is the bottleneck of bioremediation.

Swine wastewater mainly includes pig urine, partial pig manure, and piggery flushing water. These types of wastewater include high concentrations of organic matter (biochemical oxygen demand 4.5-8.0 g/L), ammonia nitrogen (1.3-1.5 g/L), and total nitrogen (1.6-2.2 g/L)^10^. It is a kind of organic wastewater which is difficult to treat. If discharged directly into local bodies of water, it will cause enormous environmental pollution. Dealing with the discharge according to standards is very difficult and costly. If the wastewater can be used as a medium for remediation of contaminated soil, not only can the cost of remediation be greatly reduced but also the wastewater can be recycled, and the pollution to the environment can be reduced.

Two strains (*Bacillus subtilis* CICC 21312 and *Candida bombicola* ATCC 22214) had good oil decomposing ability. Accordingly, they were used for bioremediation of simulated oil-contaminated soil in the present study. Swine wastewater was used instead of a nitrogen source and process water for bioremediation. The optimal ratio of strains and the optimal medium for mixed strains were determined. In addition, the soil changes after bioremediation were studied to understand physicochemical properties, enzyme activities, and microbial population, which provided technical reference and theoretical support for the field application of bioremediation in DongYing oil-contaminated soil.

## Results

### Comparison of oil degradation rates among different strains and indigenous microorganisms

The main component of petroleum pollutants in soil is polycyclic aromatic hydrocarbons (PAHs), yet the solubility of PAHs in soil aqueous solution is very low, and PAHs can be adsorbed on soil particles, resulting in poor bioavailability. This retards the biodegradation rate of PAHs at the solid-liquid interface of soil^11^. Surfactant is a kind of macromolecule with both hydrophilic and hydrophobic groups. It can increase the contact area between PAHs and soil microorganisms, improving the availability and degradation rate by curling, solubilisation, and elution^12^. However, exogenous surfactants increase the cost of soil remediation. Therefore, a strain producing sophorolipid(strain B) was selected as the soil remediation strain in this study. At the same time, sophorolipid is degradable and does not easily cause secondary pollution.

**Figure 1:**
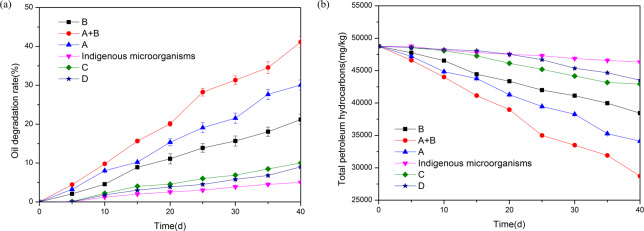
Comparison of oil degradation rates among different strains and indigenous microorganisms (▲: A; ▼: Indigenous microorganisms; ●: A + B; ■: B; ♦: C; ★: D); (**a**) Oil degradation rate (%); (**b**) Total petroleum hydrocarbons (mg/kg).

In order to select better oil degrading bacteria, four strains were selected for comparison. The comparison of oil degradation rates among different strains and indigenous microorganisms is shown in Fig. 1. The results shows that A and B have high oil degradation rate. The oil degradation rate of C and D is relatively low, so no further study is required. Because the effects of A and B are high, the synergistic effect of A and B is also investigated. The sequence of oil degradation rate of strains is A + B > A (30.08 ± 1.08%)> B (21.19 ± 1.30%), which are higher than indigenous microorganisms. After 40 d culture of strain A + B, the oil degradation rate was 41.12 ± 1.50%, while the oil degradation rate of indigenous microorganisms after 40 d was only 5.02 ± 0.15%. The main reason for the treatment effect of the mixed bacteria being better than that of the single bacterium was that strain B was a sophorolipid-producing bacteria, which could degrade PAHs well. Moreover, there was no competition between strain B and strain A, which could be mixed cultured. Strain A degraded alkane, and strain B degraded PAHs. They played a complementary role, so the degradation rate reached the optimal value.

### Construction of dominant flora and determination of the optimal ratio

**Figure 2:**
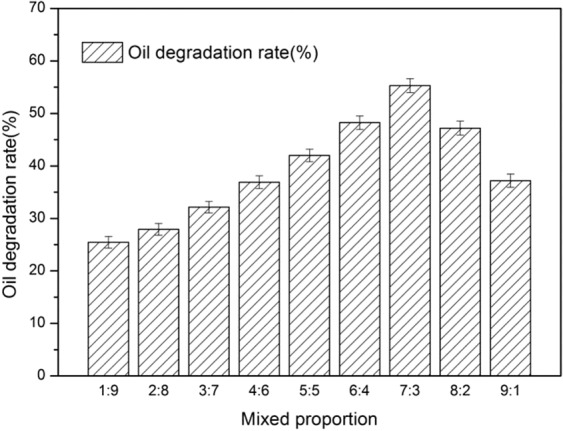
Effect of mixed proportion (A,B) on the oil degradation rate.

The effect of mixed proportions (A:B) on oil degradation rate were investigated; the results are shown in Fig. 2.

As can be seen, the proportion of strains affected the oil degradation rate. A ratio that was too low or too high reduced the oil degradation rate. The maximum oil degradation rate was 55.31 ± 1.32% at 7:3.

### Box-Behnken design and response surface analysis

The best level of the three factors (inoculation amount of mixed strains, the amount of wheat bran and the amount of swine wastewater) was determined by BBD. The factor levels used in the BBD are shown in Table 1, and results are in Table 2. Variance for the quadratic design is in Table 3.

**Table 1:** Levels of factors used in the BBD.

Factors	Level			
	−1	0		1
E: Inoculation amount(mL/kg)	90	100	110	
F: Wheat bran (g/kg)	130	150	170	
G: Swine wastewater(mL/kg)	200	230	260

**Table 2:** BBD experiments design matrix and results of oil degradation rate.

Code	E	F	G	Oil degradation rate (%)
1	−1	−1	0	52.27 ± 0.23
2	1	−1	0	52.79 ± 0.21
3	−1	1	0	62.57 ± 0.21
4	1	1	0	62.78 ± 0.29
5	−1	0	−1	62.02 ± 0.21
6	1	0	−1	51.11 ± 0.22
7	−1	0	1	62.13 ± 0.23
8	1	0	1	57.95 ± 0.26
9	0	−1	−1	52.95 ± 0.26
10	0	1	−1	57.59 ± 0.21
11	0	−1	1	52.51 ± 0.23
12	0	1	1	59.38 ± 0.27
13	0	0	0	67.73 ± 0.24
14	0	0	0	63.81 ± 0.28
15	0	0	0	66.33 ± 0.25
16	0	0	0	65.11 ± 0.21
17	0	0	0	62.98 ± 0.21

**Table 3:** ANOVA of RSM for oil degradation rate.

Source	Sum of Squares	Mean Square	F Value	Prob(P) > F
Model	416.11	46.23	5.45	0.0180
E	25.78	25.78	3.04	0.1248
F	126.41	126.41	14.90	0.0062
G	8.61	8.61	1.02	0.3473
EF	0.024	0.024	2.832E-003	0. 9590
EG	11.32	11.32	1.33	0.2859
FG	1.24	1.24	0.15	0.7132
E^2^	25.22	25.22	2.97	0.1284
F^2^	111.34	111.34	13.12	0.0085
G^2^ Residual Lack of Fit	83.09 59.39 44.84	83.09 8.48 14.95	9.79 4.11	0.0166 0.1028
Pure Error	14.55	3.64		
Cor Total	475.49			

Values of “Prob> F” less than 0.0500 indicate model terms are significant. In this case F, F^2^, and G^2^ are significant model terms. The final coding factor formula is as follows:

Oil degradation rate = 65.19 – 1.79 × E + 3.98 × F + 1.04 × G – 0.078 × E × F + 1.68 × E × G + 0.56 × F × G – 2.45×E^2^ – 5.14 × F^2^ – 4.44 × G^2^ (1) where Y is oil degradation rate, E is the inoculation amount, F is the amount of wheat bran, and G is the amount of swine wastewater.

F test was used to judge the significance of each variable in the regression equation to the response value. The smaller the probability of P, the higher the corresponding variable. The first order term F (P < 0.05) of model (1) was significant, while E and G (P > 0.05) had no significant effects. The effects of secondary order item F^2^, G^2^ (P < 0.05) was significant, and E^2^ (P > 0.05) had no significant effects. There was no significant difference between EF, EG, and FG (P > 0.05).

These results clearly showed that experimental values had a linear distribution and a good correlation (R^2^ = 0.9912). Therefore, the model could be used to predict the degradation rate of crude oil in the variable range. The maximum oil degradation rate of 68.22% was obtained at 96.53 mL/kg inoculation, 157.85 g/kg wheat bran and 232.29 mL/kg swine wastewater. To validate this prediction, three independent experiments were carried out, and an oil degradation rate of 68.27 ± 0.71% was obtained via 97 mL/kg inoculation, 158 g/kg wheat bran and 232 mL/kg swine wastewater.

**Figure 3:**
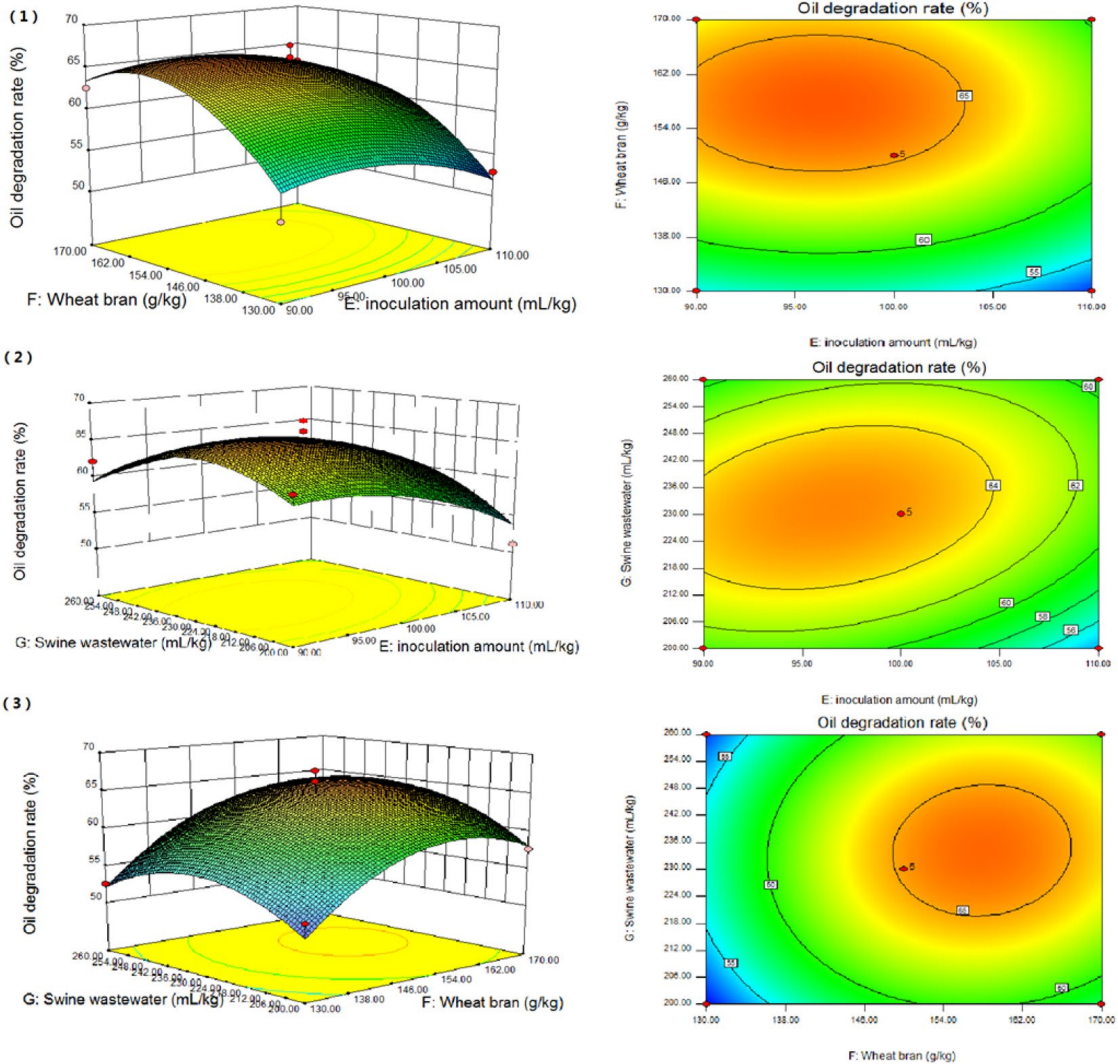
Surface and contour plots of mutual-influence. (1) effect of inoculation amount (**E**) and wheat bran (**F**); (2) effect of inoculation amount (**E**) and swine wastewater (**G**); and (3) effect of wheat bran (**F**) and swine wastewater (**G**).

The effects of the inoculation amount of mixed strains, amount of wheat bran and amount of swine wastewater on oil degradation rate were analysed by RSM. As shown in Fig. 3, three-dimensional response surface plots and contour plots were generated. This allows for studying the interaction of any two variables on the response. From the observation of the response surface graph of the interaction between the two factors, there was a strong interaction among the factors, and it was found that the steeper the curve trend, the more significant the influence. The smoother the curve trend, the smaller the influence. The shape of the contour plot represents the strength of interaction. The contour plot was round, indicating that the interaction between the two factors was weak. Moreoeer, the contour plot was oval, indicating that the interaction between the two factors was strong. Comparing the highest points and contours of the response surface in Fig. 3, there were extreme values in the selected range.

### Comparison of soil properties before and after bioremediation

Oil-contaminated soil, oil contaminated soil treated by indigenous microbial aeration composting, and oil-contaminated soil treated by microbial consortium (optimal ratio and optimal culture conditions) were recorded as M1, M2, M3, respectively. The changes of the soil’s physical and chemical properties, enzyme activities, and microbial population before and after bioremediation were investigated. The results are shown in Table 4.

**Table 4:** Properties of different types of oil contaminated soil.

Index	M1	M2	M3
Oil degradation rate ∕ %	0	5.02 ± 0.27	68.27 ± 0.71
TPH/(mg·kg^−1^)	48763 ± 254	46315 ± 231	15473 ± 99
PAHs/(mg·kg^−1^)	10281 ± 157	9754 ± 118	7199 ± 93
Alkane/(mg·kg^−1^)	26896 ± 237	25021 ± 169	18827 ± 171
pH	8.34 ± 0.32	7.98 ± 0.28	7.24 ± 0.22
C content ∕ %	65.89 ± 1.51	115.26 ± 2.15	158.41 ± 1.98
N content ∕ %	0.59 ± 0.02	1.43 ± 0.05	2.02 ± 0.05
P content ∕ %	0.59 ± 0.01	0.59 ± 0.01	0.76 ± 0.01
Dehydrogenase activity (mg·g^−1^·h^−1^)	28.01 ± 0.84	59.89 ± 2.74	86.62 ± 2.44
Catalase activity (mg·g^−1^·h^−1^)	0.90 ± 0.03	1.71 ± 0.05	3.19 ± 0.11
Polyphenol oxidase activity (mg·g^−1^·h^−1^)	1.30 ± 0.05	0.45 ± 0.01	0.32 ± 0.01
Urease activity ∕(mg·g^−1^·h^−1^)	0.031 ± 0.001	0.041 ± 0.001	0.072 ± 0.002
Bacteria counts ∕(CFU•g^−1^)	(1.21 ± 0.04) × 10^6^	(5.60 ± 0.23) × 10^6^	(7.30 ± 0.70) × 10^7^
Actinomycetes counts ∕(CFU•g^−1^)	(2.17 ± 0.15) × 10^5^	(1.23 ± 0.04) × 10^6^	(6.16 ± 0.30) × 10^6^
Fungi counts ∕(CFU•g^−1^)	(2.01 ± 0.11) × 10^4^	(7.09 ± 0.31) × 10^4^	(4.50 ± 0.29) × 10^5^

After comparing three kinds of soil samples, M3 had the best recovery effect, and the oil degradation rate reached 68.27 ± 0.71%. The oil degradation rate of M2 was only 5.02 ± 0.27%. Large amounts of nutrients were put into the sample in the process of M3 repair, so the content of organics and total nitrogen in the soil were greatly improved, while the changes in the corresponding content in M2 were not as obvious as that in M3.

The activities of dehydrogenase and catalase in M3 were the highest. This was due to the large amount of soil microorganism reproduction during the repair process, and the microorganism participated in the degradation process of oil hydrocarbon, which improved the activity of dehydrogenase and catalase. The urease activity in M3 was the highest, indicating that the soil had a strong nitrogen conversion ability, which was beneficial to the growth of microorganisms in the soil. It also provided sufficient nutritional conditions for the microorganism to participate in the degradation of oil hydrocarbon. However, the activities of various enzymes in M2 were not as obvious as those in M3.

Bacteria counts, actinomycetes counts, and fungi counts in M3 were significantly higher than that of untreated soil sample M1 and soil sample M2 after ordinary composting, which were close to the actual microbial number in the natural world.

## Discussion

The remediation of crude oil contaminated soil is a worldwide problem. Although there exist many remediation methods, this technology is still a promising remediation method. The comparison of different method was shown in Table 5. The order of oil degradation rate of different methods saw this study as better than Besalatpour et al. which was better than Wu et al. In this study, the remediation time was the shortest, and the problem of piggery wastewater pollution was solved. Moreover, it had the following advantages: (1) The treatment cost was low. In the process of treatment, all materials except strains were wastes. (2) The process could offset the disposal costs of agricultural wastes. (3) No exogenous chemical surfactants were needed during the remediation process, because strain B produced sophorolipids. (4) Swine wastewater could be a replacement for process water in bioremediation.

**Table 5:** Comparison of different methods in bioremediation of oil contaminated soil.

Method	Strain	Oil degradation rate (%)	Treating with waste	Time (d)	Reference
Land tillage	Indigenous microorganisms	50	No	120	7
Microbial consortium	Twelve varieties of bacteria	42.3	No	91	9
Microbial consortium	Two varieties of bacteria	68.27 ± 0.71%	Yes (swine wastewater)	40	This study

The main reason for the lack of consideration of the effect of aeration on microflora was as follows: although many microorganisms were aerobic, they could not be supplied with oxygen in the remediation of oil-contaminated soils. It increased the processing cost to an unaffordable level. Two highly-efficient oil-degrading strains preserved in the laboratory could improve the effect of bioremediation of oil contaminated soil. The recovery efficiency of single strain in 40 d repair process was much higher than that of indigenous microorganisms. The order of oil degradation rate of two strains was A + B > A > B. The combination of A and B had the best oil degradation rate. The best ratio (mass ratio) of the two strains was 7:3. Under this condition, the oil degradation rate of soil reached 55.31 ± 1.32%. Swine wastewater could be a replacement for nitrogen source and process water for bioremediation. The optimal medium was microbial dosage of 97 mL/kg, wheat bran of 158 g/kg and swine wastewater of 232 mL/kg. Under the optimal medium, the oil degradation rate reached 68.27 ± 0.71% after 40 d. The fertility of oil-contaminated soil, which was repaired by microbial consortium, increased significantly. In addition, the activities of dehydrogenase, peroxidase, and urease increased, and the number of microbes increased significantly. These investigations may lay a foundation for reducing the pollution of agricultural wastes, exploring a late model for bioremediation of oil-contaminated soil. In the future experiments, this microbial consortium will be studied to repair the oil-contaminated soil in other places.

## Materials and Methods

### Soil samples

Crude oil was obtained from DongYing refinery, China. The characteristic parameters of the crude oil were 18.5% polycyclic aromatic hydrocarbon and 48.4% alkane. Soil samples were collected from the unpolluted shallow (5~25 cm) soil in DongYing, China. Soil samples were broken, cleaned, mixed and sieved. According to the ratio of crude oil to soil mass ratio (1:20), the oil contaminated soil was made ready for use. Next, soil was sealed in the sterilised kraft paper bag, maintained in cold storage^13^.

### Swine wastewater

The swine wastewater used in this study was effluent coming from ZhengBang pig farm in DongYing, China. The characteristic parameters of the swine wastewater were as follows: 15.4 ± 0.2 g/L chemical oxygen demand (COD_Cr_), 6.5 ± 0.2 g/L biochemical oxygen demand (BOD_5_), 883 ± 12 mg/L suspended solid (SS), 1.1 ± 0.1 g/L ammonia nitrogen (NH_3_-N), 1.8 ± 0.1 g/L total nitrogen, 40.5 ± 0.5 mg/L total phosphorus, and pH of 7.5 ± 0.2. The wastewater was autoclaved for 20 min at 121 °C.

### Strains

*Bacillus subtilis* X-12(A) was purchased from the China Center of Industrial Culture Collection (CICC) as CICC 21312. *Candida bombicola* C-15(B) was purchased from the American Type Culture Collection (ATCC) as ATCC 22214. Strains were maintained on LB medium or YPD medium at 4 °C. *Pseudomonas aeruginosa* A-21(C) was purchased from the CICC as CICC 10204. *Arthrobacter sp*.D-2 (D) was purchased from the CICC as CICC 10758.

### Media

LB medium: yeast extract, 5 g/L; peptone, 10 g/L; sodium chloride, 5 g/L; and agar 18 g/L.

YPD medium: yeast extract, 10 g/L; peptone, 20 g/L; glucose, 20 g/L; and agar 18 g/L.

Seed medium: yeast extract, 10 g/L; peptone, 20 g/L; and glucose, 20 g/L.

All media were autoclaved for 20 min at 121 °C.

### Comparison of oil degradation rates between two strains and indigenous microorganisms

Two strains were actived in seed medium. The concentration of microbial cells was 1.5×10^8^CFU/mL. The oil contaminated soil was packed into 48 conical bottles (50 g each). The conical flasks were divided into three groups, with 16 flasks in each group. Then, 50 mL/kg of strain A and strain B suspension were added to two groups, and put in the incubator (28 °C). They were watered and ploughed every day to keep the water content around 20%. The remaining group (control group) did not receive strain suspension for investigating the effect of indigenous microorganisms. Samples were taken at intervals, and the oil degradation rate was calculatedby gravimetric method to checkthe mass fraction of oil in soil samples^14^.

### Construction of dominant flora and determination of the optimal ratio

Combinations of two strains were carried out. The mixed bacteria of different combinations were added into the oil-contaminated soil according to a certain amount of inoculation, and the total inoculation amount (50 mL/kg) of the mixed bacteria suspension in each group was equal. They were watered and ploughed every day to keep the water content around 20%. After 40 days in the incubator (28 °C), the mass fraction of oil in the soil samples was determined, and the oil degradation rate was calculated.

### Box-Behnken design and response surface analysis

Three factors affecting the oil degradation rate (inoculation amount of mixed strains, the amount of wheat bran and the amount of swine wastewater) were selected. Box-Behnken design (BBD) was used to determine optimal concentrations of factors using Design-Expert software (Version 8.0.6, Stat-Ease, Inc, USA), and to understand the relationship among various factors. The three factors were studied at three levels (Table 1), and 17 sets of experiments were carried out (Table 2). All experiments were carried out in triplicate. The optimal values of factors were obtained by analysing 3D plots. The statistical analysis of the model was represented as an analysis of variance (ANOVA).

### Analytical methods

The pH of soil samples was measured by potentiometric titration, and the volume ratio of water to soil was 2.5. The content of organic matter in soil samples was determined by potassium dichromate volumetric method. The total nitrogen content of the soil sample was determined by the semi-micro kjeldahl method. The content of total phosphorus in a soil sample was determined by the Mo-Sb antispetrophotography method^15^.

The total petroleum hydrocarbons (TPH) content in soil was determined by the gravimetric method^14^. The calculation formula for the oil degradation rate was as follows:

1 $${\rm{ODR}}\,( \% )=({{\rm{W}}}_{1}-{{\rm{W}}}_{2})/{{\rm{W}}}_{1}\times 100 \% $$

where ODR was the oil degradation rate (%), W_1_ was the content of petroleum hydrocarbon in the soil before degradation, and W_2_ was the content of petroleum hydrocarbon in the soil after degradation.

The amount of polycyclic aromatic hydrocarbon and alkane in soil were measured by the gravimetric method^14^.

Total nitrogen, total phosphorus, suspended solid, NH_3_-N, BOD_5_, and COD_Cr_ in the swine wastewater were measured according to the procedure described in standard methods for the examination of water and wastewater^15^.

The activity of dehydrogenase was determined by using triphenyltetrazolium chloride as hydrogen acceptor, which was reduced to form red formazan. It was determined by colorimetry. The amount of formazan produced in 1 g of dry soil for 6 h was an active unit of dehydrogenase. The urease activity in the soil sample was determined by Nessler’s reagent colorimetry. The amount of NH_3_-N produced in 24 h in 1 g of dry soil was an activity unit of urease. Polyphenol oxidase activity in a soil sample was determined by pyrogallol colorimetry. The amount of gallic acid produced in 3 h in 1 g of dry soil was taken as an activity unit of polyphenol oxidase. Catalase activity in a soil sample was determined by Potassium Permanganate titration. The enzyme activity was expressed by the volume number of potassium permanganate consumed in 1 g dry soil in 1h^16^. The amount of microbial population in the soil was determined by colony forming unit^17^.
